# Effects of Low-Level Blast Exposure on the Nervous System: Is There Really a Controversy?

**DOI:** 10.3389/fneur.2014.00269

**Published:** 2014-12-19

**Authors:** Gregory A. Elder, James R. Stone, Stephen T. Ahlers

**Affiliations:** ^1^Neurology Service, James J. Peters Department of Veterans Affairs Medical Center, Bronx, NY, USA; ^2^Department of Psychiatry, Icahn School of Medicine at Mount Sinai, New York, NY, USA; ^3^Department of Neurology, Icahn School of Medicine at Mount Sinai, New York, NY, USA; ^4^Friedman Brain Institute, Icahn School of Medicine at Mount Sinai, New York, NY, USA; ^5^Department of Radiology, University of Virginia, Charlottesville, VA, USA; ^6^Department of Neurosurgery, University of Virginia, Charlottesville, VA, USA; ^7^Department of Neurotrauma, Operational and Undersea Medicine Directorate, Naval Medical Research Center, Silver Spring, MD, USA

**Keywords:** animal models, blast, human studies, post-traumatic stress disorder, traumatic brain injury

## Abstract

High-pressure blast waves can cause extensive CNS injury in human beings. However, in combat settings, such as Iraq and Afghanistan, lower level exposures associated with mild traumatic brain injury (mTBI) or subclinical exposure have been much more common. Yet controversy exists concerning what traits can be attributed to low-level blast, in large part due to the difficulty of distinguishing blast-related mTBI from post-traumatic stress disorder (PTSD). We describe how TBI is defined in human beings and the problems posed in using current definitions to recognize blast-related mTBI. We next consider the problem of applying definitions of human mTBI to animal models, in particular that TBI severity in human beings is defined in relation to alteration of consciousness at the time of injury, which typically cannot be assessed in animals. However, based on outcome assessments, a condition of “low-level” blast exposure can be defined in animals that likely approximates human mTBI or subclinical exposure. We review blast injury modeling in animals noting that inconsistencies in experimental approach have contributed to uncertainty over the effects of low-level blast. Yet, animal studies show that low-level blast pressure waves are transmitted to the brain. In brain, low-level blast exposures cause behavioral, biochemical, pathological, and physiological effects on the nervous system including the induction of PTSD-related behavioral traits in the absence of a psychological stressor. We review the relationship of blast exposure to chronic neurodegenerative diseases noting the paradoxical lowering of Abeta by blast, which along with other observations suggest that blast-related TBI is pathophysiologically distinct from non-blast TBI. Human neuroimaging studies show that blast-related mTBI is associated with a variety of chronic effects that are unlikely to be explained by co-morbid PTSD. We conclude that abundant evidence supports low-level blast as having long-term effects on the nervous system.

## Introduction

While an uncommon cause of traumatic brain injury (TBI) in civilian life (1), blast-related TBI has been of long-standing interest in military head trauma. During World War I, it was first recognized that blast exposure could be associated with psychological and neurological symptoms that were reminiscent of both the post-concussion syndrome and what would now be called post-traumatic stress disorder (PTSD) (2–9). Recently, there has been renewed interest in blast-related TBI because of its frequency in the conflicts in Iraq and Afghanistan (8, 10–32). Indeed, estimates are that 10–20% of veterans returning from these conflicts have suffered a TBI with blast exposure most commonly related to improvised explosive devices (IEDs) being the most frequently attributed cause (10, 33).

Most attention focused initially on the moderate to severe end of the TBI spectrum (34), the type of TBI that would be recognized in theater and the war in Iraq has resulted in the highest number of service-related severe TBIs since the Vietnam era (35). However, what soon became apparent was that many returning veterans began appearing at Veterans Affairs (VA) Hospitals and other facilities with symptoms suggestive of the residual effects of mild TBIs (mTBIs) that were never recognized prior to discharge. In fact, mTBIs vastly outnumber moderate to severe TBIs in this population (10, 33).

This renewed interest has lead to a rapid expansion of clinical as well as animal studies related to blast. Despite these efforts, questions remain concerning how blast exerts its effects on the nervous system and even what effects can reasonably be attributed to blast. Indeed, a recent Institute of Medicine report (36) concluded that in terms of long-term adverse health outcomes in human beings, there was sufficient evidence of a causal relationship to blast only for penetrating eye injuries and some long-term effects on the genitourinary system. For post-concussion symptoms and persistent headaches following blast-related mTBI, the report concluded that there was only sufficient evidence for an association (36).

Questions have also emerged as to whether pathophysiologically blast-related TBI is different from the type of non-blast TBI (nbTBI) typical of civilian trauma where injury is caused by inertial and rotational forces along with the effects of blunt impact (37, 38). The most direct physical effects of these forces are bleeding, direct tissue damage, and mechanical shear stress along white matter tracts, which in turn leads to activation of a variety of pathophysiological cascades that are associated with further tissue damage (37, 39). Blast injuries by contrast result from a pressure wave generated at a distance and transmitted though air, which may induce stresses in the brain without significant global motions being imparted. Damage to the nervous system is thought to occur through biophysical mechanisms related to the traveling shock wave’s interaction with the brain (40–42), although it has also been suggested that a blast wave striking the body can induce oscillating pressure waves, which can be transmitted through the systemic circulation to the brain (12, 43).

Blast injuries have typically been divided into four or five categories (13, 19, 44). Injuries directly related to the blast wave are referred to as primary blast injuries. The blast explosion can propel objects including shrapnel contained within the IED causing what is referred to as secondary injury. The blast wave may also cause victims to be knocked down or thrown into solid objects resulting in what is called tertiary injury. Quaternary injuries comprise a group of miscellaneous injuries not classifiable into other categories including burns or the effects of inhaling noxious gases. Finally, the term quinary injury is sometimes used to refer to illnesses that result from chemical, biologic, or radiologic substances released during an explosion (19). Development of this blast taxonomy began during World War II (45) and remains useful today. However, it does not encompass all injury mechanisms encountered today such as platform shock acceleration where energy is transferred through the ground or a vehicular structure (46). This phenomenon, which is particularly relevant to the common situation where injury occurs to personnel in a vehicle hit by an IED occurs due to complex mechanisms unrelated to the primary blast wave itself (44).

Close exposure to a high-pressure blast wave can cause extensive CNS injuries in human beings with high-level blast exposures appearing to be particularly prone to inducing hemorrhagic lesions (47). High-level blast exposures in animals can also produce a variety of gross as well as histological pathology (11, 26). However, the relative importance of the blast injury mechanisms discussed above varies depending on the severity of injury. Blast injuries severe enough to cause moderate to severe TBI are without doubt a mix of secondary and tertiary injuries combined with the contribution of the primary blast wave. In both human beings and animals, secondary and tertiary injuries likely activate many of the same pathophysiological cascades seen in nbTBI. What is less clear is how the primary blast wave damages the brain. Blast-related mTBI differs in that secondary and tertiary injuries are less prominent with most cases presumed to reflect mostly the direct effects of the primary blast wave.

## Defining Blast-Related mTBI in Human Beings

Blast-related TBI is currently defined using the same criteria used for defining nbTBI. In human beings, TBI severity is defined in relation to the degree of neurological dysfunction at the time of injury. TBI severity is thus considered separate from TBI outcome, i.e., the chronic signs and symptoms as well as functional impairment that may persist after a TBI. TBI also requires an event. In the case of nbTBI, the head is struck or strikes an object or in blast-related TBI is enveloped by the blast overpressure wave. Historically, TBI has been graded as mild, moderate, or severe based primarily on the immediate effects of the TBI on consciousness.

The first widely used staging system for TBI was the Glasgow Coma Scale (48), which divides TBIs into mild, moderate, and severe based on three clinical criteria: eye opening, best motor, and best verbal response (48–50). Subsequently, several classifications have been proposed (51–54). Four of the more commonly used criteria are those developed by the American Congress of Rehabilitation Medicine (51), the Centers for Disease Control and Prevention (CDC) (52), the World Health Organization (WHO) (53), and the Department of Defense (DoD)/Department of Veterans Affairs (54). The features of these are summarized in Table 1.

**Table 1:** **Consensus criteria for the classification of human mTBI**.

	American Congress of Rehabilitation Medicine (51)	Centers for Disease Control and Prevention (52)	World Health Organization (WHO) (53)	Department of Defense/Department of Veterans Affairs (54)
Alteration of consciousness	Any alteration of mental state at the time of the accident (e.g., feeling dazed disoriented or confused).	Any period of observed or self-reported transient confusion, disorientation, or impaired consciousness; any period of observed or self-reported dysfunction of memory (amnesia) around the time of injury.	Confusion or disorientation for 30 min or less.	A moment up to 24 h.
Loss of consciousness (LOC)	Any period of less than 30 min.	Any period of observed or self-reported loss of consciousness lasting 30 min or less.	30 min or less.	0–30 min
Post-traumatic amnesia (PTA)	Any loss of memory for events immediately before or after the accident but not greater than 24 h.	Post-traumatic amnesia less than 24 h.	Post-traumatic amnesia for less than 24 h.	0–1 day.
Glasgow coma scale (GCS)	13–15 at 30 min post-injury.	13–15 as assessed by a qualified healthcare provider at the first opportunity.	13–15 at 30 min post-injury or later upon presentation for healthcare.	13–15
Focal neurological signs	Focal neurological deficits that may or may not be transient allowed as long as other conditions are met.	Focal neurological deficits (e.g., hemiplegia) allowed if other criteria are met.	Transient neurological abnormalities such as focal signs, seizures, allowed.	
Brain imaging		Focal lesions on neuroimaging (e.g., computed tomography) allowed if other criteria are met.	Intracranial lesions allowed as along as not requiring surgery.	Routine brain imaging must be normal.
Other		Other neurological or neuropsychological dysfunction, such as seizures acutely following head injury allowed; penetrating craniocerebral injury excluded.	Effects not due to drugs, alcohol, medications, caused by other injuries or treatment for other injuries (e.g., systemic injuries, facial injuries, or intubation); not caused by other problems (e.g., psychological trauma, language barrier or coexisting medical conditions) or caused by penetrating craniocerebral injury.	

All use alteration or loss of consciousness as the primary indicator that a TBI occurred. This alteration may range from any transient alteration of consciousness including being as little as momentarily dazed or confused up to loss of consciousness for 30 min. All incorporate the Glasgow Coma Scale and exclude cases with scores less than 13 as well as allow for a period of post-traumatic amnesia lasting up to 24 h. The CDC criteria include pre-injury as well as post-injury amnesia, while the DoD/VA and WHO criteria only consider post-traumatic amnesia. The American Congress of Rehabilitation Medicine, CDC, and WHO guidelines allow focal neurological deficits to be present as long as other criteria are met while focal symptoms or signs are not mentioned in the DoD/VA guidelines. Brain imaging results are not considered as a factor in the American Congress of Rehabilitation Medicine guidelines. The CDC and WHO guidelines allow focal lesions on brain imaging if other criteria are met or in the case of the WHO guidelines if surgery is not required. By contrast, the DoD/VA guidelines state that routine neuroimaging must be normal. In the CDC guidelines, penetrating craniocerebral injury is excluded and the WHO guidelines add the stipulation that any neurological dysfunction must not be due to drugs, alcohol, medications, or be caused by other injuries, treatments, psychological trauma or other factors that could confound diagnosis.

Thus, while there is no universally accepted definition of mTBI in human beings, all four sets of criteria use some level of alteration or loss of consciousness as the primary indicator that a TBI occurred. However, the definitions encompass a wide range of severity ranging from as little as being momentarily dazed or confused up to loss of consciousness for 30 min. Three of the four allow focal findings on exam or focal lesions on imaging as long as other criteria for mTBI are met. Inclusion of such cases broadens the mTBI spectrum. Indeed, other criteria separate off into a category labeled “complicated” mTBI that subset of patients who could clinically be classified as mTBI but in whom neuroimaging reveals focal intracranial lesions (55). While the concept of complicated mTBI has not made its way into the major consensus criteria, a number of studies suggest that complicated mTBI is associated with more prolonged recovery and residual neurocognitive impairments (56–66). While a variety of ancillary studies including advanced neuroimaging techniques and serum markers such as S-100 protein have been investigated as markers of brain injury severity (67, 68), none has gained general acceptance as diagnostic markers of mTBI.

## Difficulties in Applying Current Definitions of mTBI to Blast Injury

Within the TBI model, blast exposures are dichotomized into TBI or not TBI i.e., either an event has occurred that can be labeled a “TBI” or it has not. If there is no TBI event, then it is concluded that no clinically significant blast exposure occurred. The first difficulty posed by this approach is the defining of a TBI event. The term “concussion” is today treated as being largely synonymous with mTBI. Yet concussion once required a transient loss of consciousness as part of its definition. However, largely due to research in the sports medicine literature (69), it has become clear that loss of consciousness is not necessary for a clinically significant TBI event to have occurred. Thus, current definitions of mTBI, as discussed above, include even the most transient alteration of consciousness including being as little as momentarily dazed, confused, or “seeing stars.” These newer criteria greatly expand the definition of mTBI and most blast-related mTBIs in veterans returning from Iraq and Afghanistan do not involve loss of consciousness (10, 70–73).

Expanding the mTBI definition represents progress if it captures a larger spectrum of clinically significant blast exposures. However, it may be misleading, if it labels clinically insignificant blast exposures as TBI as suggested by some of the TBI/PTSD overlap literature discussed below. Indeed, the expanded definition can sometimes make deciding whether a mTBI has occurred difficult, which is often done based on histories obtained long after an event that occurred in settings where it may be difficult to separate a transient neurological disturbance from a psychologically based stress reaction.

Current definitions of mTBI pose another problem in that if an exposure just sufficient to produce the most minimal alteration of neurological functioning can be considered clinically significant then could exposures beneath the threshold to produce a TBI event matter clinically as well. This issue seems particularly relevant to blast due to the nature of blast exposure that involves a blast wave propagated for considerable distances through air thereby exposing large areas to a continuous range of air-shock conditions. If subclinical blast exposure affects the brain, then any definition of TBI requiring a transient neurological disturbance would fail to capture the full spectrum of blast effects and indeed would suggest that the TBI model may fail to capture a wider spectrum of blast-related brain injury.

Indeed, under such a scenario, multiple subclinical exposures might matter more than a single exposure sufficient to be identified as a TBI event. Multiple blast exposures have been common in the war zones in Iraq and Afghanistan even in subjects not known to have suffered a TBI. For example, in the study of Hoge et al. (10) over 55% of non-injured controls reported two or more occasions in which they were near an IED explosion suggesting that most controls in these settings have been subjected to blast exposure. Comparisons in multivariate regressions are performed along a dichotomy of TBI vs. no TBI. However, if a continuous gradient of subclinical exposures matter, especially cumulative exposures just below the TBI threshold then the dichotomy may not be real and cumulative exposure may matter more.

## Separating Blast-Related mTBI and PTSD

One of the striking features of the mTBI cases being seen in veterans from Iraq and Afghanistan is the frequent presence of PTSD (11). Indeed, PTSD or depression is present in over one-third of Iraq veterans with suspected post-concussion syndromes secondary to mTBI (10, 74). The frequent presence of PTSD has complicated diagnosis since the clinical distinction between a post-concussion syndrome and PTSD is often difficult with the two disorders having many overlapping symptoms (11). In both somatic complaints including fatigue, irritability and poor sleep are frequent. Impaired concentration, attention, and memory are also common, and neuropsychological test profiles can look similar with deficits in attention, working memory, executive functioning, and episodic memory present in both (74).

The problem of distinguishing the two disorders is not new and has roots dating back to the entity first recognized during World War I known as “shell shock” (2–8). During this era, a debate raged concerning whether shell shock was a physical injury or the result of psychic trauma. The debate ended without decisive resolution but with the British government clearly favoring psychological over organic explanations. Indeed, the 1922 Southborough report of the War Office’s Committee of Enquiry into “Shell-Shock” concluded that regular units with high morale were virtually immune from shell shock (75). At the beginning of World War II, the British government suppressed the use of the term “shell shock” (76). However, soldiers continued to be exposed to blasts and to present with a similar range of symptoms. The controversy regarding physical vs. psychological injury continued again without any clear resolution (2). Following the Vietnam War, PTSD became conceptualized as a diagnostic entity and was added to the third edition (DSM-III) of the Diagnostic and Statistical Manual of Mental Disorders of the American Psychiatric Association (APA) (77). The role of blast exposure in this symptom complex remerged in the popular press and scientific literature following the onset of the conflicts in Iraq and Afghanistan (78). While similar in many ways to the war exposures of the past, blast exposures in the most recent conflicts have differed in first their high prevalence. Indeed, it has been estimated that 75% of casualties in Iraq and Afghanistan have been due to explosions (36). Improved personal protective equipment seems to have mitigated in particular the severity of blast-related lung injuries. Such factors along with improved frontline medical care have lead to a lower percentage of blast fatalities and thus increased the number of survivors who may live to experience the effects of blast-related TBI.

TBI and PTSD have an interesting relationship in that the two disorders can be considered different ends of a spectrum with TBI being the classic example of an organic brain disease and PTSD a psychologically based reaction to a stressor that was not associated with physical injury. Indeed, it has been suggested that the post-traumatic amnesia associated with TBI may protect against the development of PTSD, based on the notion that amnesia for the event precludes formation of the core affective responses needed to develop PTSD (79). While PTSD can clearly develop after even moderate to severe TBI, evidence does suggest that PTSD rates are higher in subjects who remember the TBI incident compared to those with no memory for the event (80, 81).

The association of PTSD with mTBI might be explained by dual exposures to PTSD stressors as well as independent TBI events. However, other studies have suggested that the apparent epidemic of mTBI in veterans from Iraq and Afghanistan may be an artifact of current definitions of mTBI, which lower the threshold for diagnosing an mTBI to the most transient alteration of consciousness. The study of Hoge et al. (10) first raised such questions. They surveyed over 2700 U.S. Army infantry soldiers from two brigades, 3–4 months after returning from a 1-year deployment in Iraq. In this study, the most frequent cause of TBI was blast and all but 4 of 384 TBIs were mTBI. Among the mTBI, 5% reported loss of consciousness and 10% occurred without loss of consciousness. In soldiers who reported an mTBI complaints of headache, poor memory and concentration were frequent suggesting that a persistent post-concussion syndrome was present. However, using a multivariate logistic regression analysis, after adjusting for the frequent co-existence of PTSD and depression, an mTBI history was no longer associated with any post-concussion symptoms, except for headache.

Hoge et al. (82) argued later that current-screening criteria for mTBI are flawed. Multiple subsequent studies from this group as well as others have supported this interpretation suggesting that post-concussion symptoms and other adverse physical or neuropsychological outcomes following blast-related mTBI are only non-specifically related to blast, better explained by PTSD or only related to blast when there has been loss of consciousness, which is the minority of cases using current definitions of mTBI (70, 72, 73, 83–92). Indeed, the 2014 Institute of Medicine report (36) on the long-term consequences of blast injury concluded that based on human studies, there is “limited/suggestive evidence that most of the shared symptoms are accounted for by PTSD and not a direct result of TBI alone” and that for post-concussion symptoms and persistent headaches following blast-related mTBI, there was only sufficient evidence to suggest an association. While other studies suggest that a blast-related mTBI might be more than a coincidental exposure (93–100), the clear message from many studies is that much of what is presently being called post-concussion syndrome secondary to blast-related mTBI is really PTSD.

## Defining Blast-Related mTBI in Animal Models

Animal models should allow effects of primary blast to be determined free of many confounding variables present in natural human exposures (11, 26). Recently, Cernak (101) has discussed the general requirements for animal models of blast-induced neurotrauma. Developing an animal injury model involves both a choice of species as well as exposure simulation. Choice of species has practical as well as theoretical implications. Rodents, mostly rats, have been most commonly used (11, 26), a choice that without doubt has been driven more by practical than theoretical considerations. Besides availability, rodents offer advantages in the power of the genetic systems that exist particularly in mice. Rodents may be less than ideal; however, in that rodent brains lack the gyri and sulci found in human beings, a factor that likely affects the brain’s mechanical response to acceleration impulse (102). The white/gray matter ratio is also less in rodents, another factor that may affect the brain’s reaction to injury. Pigs and non-human primates have brains more similar to human beings but their cost and in the case of non-human primates, availability limits their wider use.

Animals have been exposed to various forms of blast ranging from direct exposure to live explosives to more commonly controlled blast waves produced by compressed-air generators (11, 26). Although live explosives may best model exposure in the field, this approach affords less experimental control over the physical characteristics of the blast wave as well as depending on the injury paradigm may involve difficulty in separating effects of the primary blast wave from secondary, tertiary, or even quaternary injuries. Pressure generators allow blast overpressure effects to be studied in isolation, offering more experimental control. However, conventional shock tubes can only approximate some aspects of explosive blast conditions. While they replicate the ideal blast wave, they lack the capability of modeling the non-ideal blast wave with its multiple shock and expansion fronts that occur in real-life settings.

Interpreting the shock tube literature is also complicated by there being no standardized conditions for conducting shock tube research (101, 103). Shock tube apparatus and blast insult conditions, as well as specimen mounting, degree of head restraint, and location relative to the tube can vary greatly between labs. Blast waves are characterized by their peak overpressure, duration, and impulse. Different shock tubes may produce pressure waves with differing characteristics leading to different biological effects. For example, large peak pressures delivered over short durations may be better tolerated than lower pressures experienced over long durations (103). Yet, many studies report only peak pressures making it difficult to sometimes compare studies even within species. Sundaramurthy et al. (104) have also shown that the biomechanical loading experienced by rats in a shock tube can vary widely depending on whether the animal is placed inside or outside the tube and at different locations along the length of the tube. Choice of species also affects scaling considerations important in choosing shock wave parameters (105). In addition to size, skull biomechanical properties as well as anatomy of the orbits and sinuses likely affect how external loads become imparted to the brain. Pigs for example have relatively thick skulls compared to human beings while rodents have much thinner skulls, factors that apart from size must be considered in experimental design.

Finally, it is not clear that all injury to the brain occurs through direct effects. For example, it has been suggested that blast waves hitting the thorax or abdomen can be transmitted through the vasculature to the brain causing injury (43, 106). The importance of this mechanism remains unclear. However, it is interesting that in the most recent conflicts, there has been little evidence of “blast lung” implying that the Interceptor Body Armor being provided to soldiers may be effective in mitigating blast injury to the thorax. If so, animal models allowing thoracic exposures may be reproducing effects that are no longer as relevant to current wartime exposures.

However, whatever animal model is chosen, the question arises how to define mTBI given that in human beings TBI severity is defined in relation to the degree of alteration of consciousness at the time of acute injury. While studies of experimental blast injury in animals have been conducted without anesthesia (107, 108), essentially all studies being conducted today are done under general anesthesia, which precludes observing any transient alteration of neurological functioning at the time of exposure. The problem thus becomes how to apply a definition of mTBI in human beings in which severity is judged by alteration of consciousness at the time of injury to animal studies conducted under anesthesia where alteration of consciousness at the time of injury cannot be assessed.

Thus, in animal models, injury severity can only be assessed by the residual effects observed after anesthesia has worn off, i.e., outcomes. Even if animals could be studied without anesthesia, the problem of how to operationalize being “dazed or confused” or “seeing stars” in an animal with no capacity for self-report seems formidable. Given the difficulty of applying definitions of human mTBI to animal studies, the label “mTBI” probably has only limited usefulness in animals. Indeed from here forward, we refer to animal studies thought to be the equivalent of human mTBI based on outcomes as “low-level” blast.

Outcomes that might be considered in animal models include behavioral, pathological, physiological, biochemical, or neuroimaging results. Among these, behavior and pathology are readily available in animals. However, since it is rare that mTBI patients come to postmortem exam, little is known about the pathology of mTBI especially in the acute phase (38). Modern neuroimaging may provide one of the best opportunities for comparative studies since this modality is well developed in human beings (38) and being applied more to animal models (see below).

The issue then becomes how to develop a relevant animal model of low-level blast. One approach taken by Ahlers et al. (107) was to expose animals to sequentially higher blast levels and to determine whether a threshold could be identified where outcomes would mirror those expected of a human mTBI without features inconsistent with mTBI. In these studies, which utilized the pneumatically driven shock tube at the Walter Reed Army Institute of Research (WRAIR) (107), initially unanesthetized rats were exposed to progressively higher blast exposures in different orientations at peak pressures of 36.6 kPa (associated with a duration of 4.1 ms and impulse of 75.2 kPa*ms), 74.5 kPa (duration 4.8, impulse 175.8), and 116.7 kPa (duration 6.8, impulse 335.5). Exposures up to 74.5 kPa (equivalent to 10.8 psi) lead to no persistent neurological impairments, although anterograde memory deficits were observed in rats exposed to 74.5 kPa blasts when facing the blast wave. In addition, 36.6 and 74.5 exposures produced no gross neuropathological effects and histological examination of the lung showed no hemorrhage or other abnormalities. By contrast, 116.7 kPa exposures lead to overt pathology with approximately 30% of rats having subdural hemorrhages and cortical contusions, a finding not consistent with human mTBI. All animals exposed to 116.7 kPa blasts had frequent pulmonary hemorrhages suggesting that these levels of exposure produce a state of polytrauma. Similar studies conducted using anesthetized rats in the WRAIR shock tube exposed to single or multiple blasts have also found that 36.6 and 74.5 level exposures are consistent with a low-level blast exposure (107, 109–111).

Thus, based on studies using the WRAIR shock tube, a dividing line seems to exist between 74.5 and 116.7 kPa that separates low-level blast in rats from moderate to higher level blast exposures that are more equivalent pathologically to human moderate to severe TBI in the context of polytrauma. The findings in the WRAIR studies are consistent with other investigations that have noted similar patterns of injury at pressures of 116.7 kPa and above in rats (112–115). Studies from other labs using exposures of 74.5 kPa or lower also seem largely consistent with the WRAIR findings suggesting that exposures in rats up to 74.5 kPa across a range of durations can reasonably be called low-level blast exposure (116–121). Indeed very similar to the WRAIR studies, Baalman et al. (121) found that while 74 kPa (duration ≈ 4 ms) peak pressures resulted in only subtle pathology, the next highest exposure (98 kPa) resulted in lung trauma and death.

Pressures that mimic low-level blast in other species may of course differ and require validation in relation to outcomes in those species. Of interest are several studies in mice that have examined blast overpressure exposures similar to those that appear consistent with low-level blast in rats. For example, Goldstein et al. (122) studied mice exposed to a single blast exposure of 77 kPa. However, while this exposure is within the range that might be reasonably considered low level in rats, the extent of neuropathology seen in this model seems more equivalent to higher level blast. By contrast, Huber et al. (123) studied mice exposed to 108 kPa pressures and describe what seems like a more mild pathology. In a third set of studies, mice were exposed to 68, 76, or 105 kPa exposures (124, 125). With 68 kPa exposures, scattered axonal pathology was found in multiple CNS tracts (125) with no mortality if the mice were exposed in the prone position although 5% of the mice died if exposed in the supine position. At higher exposures, there was between 11% (76 kPa) and 33% (105 kPa) mortality, if the mice were exposed in the prone position and 37% (76 kPa) to 53% (105 kPa) if exposed supine, levels of mortality that would not be expected of an mTBI exposure. These studies emphasize not only the importance of species specific differences but also that outcomes within the same species may vary with similar pressure exposures. One factor in particular differing between the studies discussed above was that Goldstein et al. (122) used no head restraint allowing the head to be subjected to significant rotational motion as well as likely focal impact injury. Notably, when Goldstein et al. (122) restricted head movement, hippocampal dependent leaning and memory deficits disappeared. Effects on pathology after head restraint were not reported (122). Restricting exposure to the head may also influence effects as Heldt et al. (126) have described a model in which mice exhibited only limited CNS pathology with exposures of 20–60 psi if the blast wave was delivered to a small area on one side of the cranium.

## Effects of Low-Level Blast Exposure in Animal Models

Most studies of blast in animals have utilized relatively high-level exposures that likely more approximate moderate to severe TBI (11, 26). However, particularly in recent years studies have appeared assessing effects of lower level blast. To search for papers reporting low-level blast exposure in animal models, we searched Pubmed using the terms “blast” and “traumatic brain injury.” Articles were selected for review based on the relevance of the title and abstract. References in the studies that were selected for review were examined for additional relevant citations.

In rats, we considered as low-level blast those studies that utilized blast exposures of ≈ 75 kPa or less based on the considerations discussed above and where sufficient pathological information was available to conclude there was no gross pathology particularly hemorrhages that would be incompatible with mTBI. Studies in mice were more difficult to judge due to there being fewer studies and the seeming variability in response to blast in the 68–108 kPa range (122–125) (although see discussion above concerning differences in these studies). However, a series of studies in mice using exposures to live explosives in the 2.5–5.5 psi range seem compatible with a definition of low-level blast (127–129).

Table 2 summarizes studies of exposures in what we term the low-level blast range. These papers first document that low-level blast exposures are transmitted to the brain (130). Chavko et al. (130) showed that pressures as low as 35 kPa are transmitted to the brain in rats although the patterns and durations of pressure traces inside the brain depended on the orientation to the blast with frontal exposures resulting in pressure traces of higher amplitude and longer duration than with other orientations. Peak pressures measured in brain were very similar to those in air (131). Interestingly, the observation of higher pressures with frontal exposures is similar to what has been observed in helmeted manikins (132). Sustained increases in intracranial pressure (ICP) have been documented for 10 h in rats exposed to 30 or 60 kPa blasts (119). While as noted above, it is difficult to directly compare exposures across species, similar observations have been made in pigs subjected to 23–45 kPa exposures from the firing of military weapons (120, 133). As in rats, peak pressures in the pig brain had similar magnitudes to those recorded in air with lower blast wave frequencies being transmitted more readily to brain than higher frequencies (120, 133).

**Table 2:** **Effects of low-level blast exposure in animal models**.

Species	Peak overpressure (shock tube unless otherwise indicated); duration and impulse given when reported	Pathological, biochemical, physiological, and imaging findings	Behavioral findings	Reference
Rat	2.8 or 20 kPa (duration ≈2 ms)	20 kPa exposure resulted in decreased performance on rotametric and grip-strength tests; scattered hyperchromatic cells visible in the cerebral cortex at 1 day or 1 week post-exposure; animals receiving aminoguanidine before or after blast protected.		(134)
Rat	40 kPa (duration 4 ms)	Sensor in third ventricle detected blast pressure wave in brain with similar magnitude to that in air.		(131)
Rat	10, 30, or 60 kPa (duration 4–6 ms)	Intracranial pressure (ICP) increases of 80–145% at 10 h post-blast at 30 and 60 kPa exposures; ICP increases less in rats fed processed cereal feed.	Morris water maze (MWZ) impaired 2 days after exposure to 10 or 30 kPa blast; no functional impairment on MWZ in rats fed processed cereal feed.	(119)
Rat	Open-field exposure (120 kg TNT) 48.9 kPa (7.1 psi; duration 14.5 ms) or 77.3 kPa (11.3 psi; duration 18.2 ms)	Cortical neurons darkened and shrunken with narrowed vasculature in cerebral cortex 1 day after blast; TUNEL-positive oligodendrocytes and astrocytes in white matter at day 1; more amyloid precursor protein immunoreactive cells in white matter; altered expression of over 5700 genes in the brain post-blast.		(118)
Rat	35 kPa (duration 4.1 ms)	Pressure wave transmitted to brain; frontal exposures (head facing blast) resulted in pressure traces of higher amplitude and longer duration than side exposure or head facing away from blast.		(130)
Mouse	Open-field explosives; 4 and 7 m distance from blast (5.5 and 2.5 psi)	Increased blood–brain barrier permeability 1 month post-blast on MRI T1 weighted images; increase in fractional anisotropy (FA) and decrease in radial diffusivity on diffusion tensor imaging (DTI); upregulation of manganese superoxide dismutase 2 in neurons and CXC-motif chemokine receptor 3 around blood vessels in fiber tracts.	Reduced preference for a novel object at 7 and 30 days post-blast; more rearing events in a staircase climbing task at 7 and 30 days post-blast; less alteration in a Y-maze task at 7 days (2.5 and 5.5 psi exposures) and 30 days (5.5 psi exposure) after blast exposure.	(127)
Rat	11.5 kPa (duration 200–250 μs)	Delayed cytoskeletal proteolysis of alpha II-spectrin in cortex and hippocampus by 12 h post-injury; cell death minimal and localized predominantly in corpus callosum and periventricular regions; evoked compound action potentials (CAP) in the corpus callosum increased in duration at 14 and 30 days post-injury with depression of unmyelinated fiber amplitudes; shielding head attenuated alpha II-spectrin cytoskeletal breakdown.		(117)
Rat	Single 36.6 kPa (duration of 4.1 ms, impulse 75.2 kPa*ms) and 74.5 kPa (duration 4.8 ms, impulse 175.8 kPa*ms); repeat (12) 36.6 kPa	No general histopathology; no evidence of axonal pathology based on APP immunohistochemical staining.	Anterograde memory deficits on a passive avoidance task after 74.5 kPa exposure; repeat exposure to 36.6 kPa produced transitory learning deficits on MWZ.	(107)
Rat	Repeat (three) 74.5 kPa (duration 4.8 ms, impulse 175.8 kPa*ms)	Elevation in the amygdala of the protein stathmin 1.	Increased anxiety, enhanced acoustic startle, and enhanced response in the contextual phase of a fear-conditioning paradigm in blast exposed; altered response to a predator scent after blast exposure.	(109)
Rat	Single 36.6 or 74.5 kPa (duration 4.8 ms, impulse 175.8 kPa*ms)	Brain Aβ levels decreased acutely following exposure; levels of APP protein increased on Western blotting although no evidence of axonal pathology based on APP immunohistochemical staining; no change in levels of β-site APP cleaving enzyme 1 (BACE1), or the γ-secretase component presenilin-1.		(135)
Rat	Single or multiple (three) 74.5 kPa (duration 4.8 ms, impulse 175.8 kPa*ms)	No general histopathology but focal cortical lesions thought to represent shear-related lesions found in many brains.		(110)
Mouse	Single live explosive detonations (2.5–5.5 psi peak overpressure).	Increased ganglioside GM2 in hippocampus, thalamus, and hypothalamus with depletion of ceramides.		(129)
Mouse	2.5 psi (17.2 kPa)	Compared hippocampal transcriptome in mice subjected to weight drop or blast injury; divergence in hippocampal transcriptome observed between models; Alzheimer’s disease-related pathways displayed a markedly different form of regulation depending on the type of TBI.	Reduced preference for a novel object at 7 and 30 days post-blast; no changes in Y-maze, passive avoidance, or elevated plus maze.	(128)
Rat	74 kPa (duration ≈4 ms)	Two weeks after exposure, little or no changes in a panel of common injury markers in cortex, corpus callosum, or hippocampus; no change in spectrin breakdown products in brain; significant shortening of the axon initial segment (AIS) in cortex and hippocampus of blast-exposed; next highest pressure (98 kPa) resulted in lung trauma and death.	Rats exposed to a blast spent less time exploring a novel object at 2 weeks post-exposure.	(121)
Rat	100 kPa (duration 0.46 ms)	Region specific decreases in fractional anisotropy on DTI in blast-exposed animals at 4 and 30 days post-exposure; evolution of DTI changes during the 4–30-day post-blast period with greater changes at 30 days.	Deficits in memory in MWZ and less activity in an open field at 4 and 30 days; no changes blast vs. control in an elevated plus maze.	(116)
Mouse	100 db noise exposure coupled with a 2 psi (duration 0.5 ms) air blast administered in sessions of 60 exposures over 1 min.		Impaired object recognition and evidence of anxiety in an elevated O-maze; following noise/blast exposure mice spent less time at the edges of an open-field chamber.	(108)
Rat	Repeat (three) 74.5 kPa (duration 4.8 ms, impulse 175.8 kPa*ms)		Blast exposure caused more rapid extinction of a conditioned fear response if the overpressure injury was administered after learning of the conditioned fear response.	(136)
Mice	20–60 psi blast exposures delivered to a focal area on one side of the cranium.		25–40 psi blast exposures produced transient anxiety in an open field; mice exposed to 50–60 psi blast exposures exhibited increased acoustic startle, perseverance of a learned fear response, an enhanced contextual fear response, depression-like behavior, and diminished prepulse inhibition.	(126)
Rat	Single or multiple (three) 74.5 kPa (duration 4.8 ms, impulse 175.8 kPa*ms)	Microvascular pathology present at 24 h after injury within an otherwise normal neuropil; chronic changes in the microvasculature evident many months after blast exposure.		(111)

Pathologically (Table 2), low-level blast exposure in rats leads to no general histopathology, although most studies have focused on the pathology most commonly seen in moderate to severe nbTBI, which may not be the most relevant to blast. Some studies have described focal cortical lesions that are likely the result of shear-related effects (110), and a selective microvascular pathology has been described at the ultrastructural level (111). Others have described scattered pyknotic neurons (118, 134) with TUNEL-positive oligodendrocytes and astrocytes in white matter and periventricular regions (117, 118). More amyloid precursor protein (APP) immunoreactive cells in the white matter have been observed (118). One study found shortening of the axon initial segment in cortex and hippocampus of blast-exposed rats (121). Biochemically, proteolysis of alpha II-spectrin in cortex and hippocampus has been observed at 12 h post-injury (117) as well as upregulation of manganese superoxide dismutase 2 (127) and increased expression of the CXC-motif chemokine receptor 3 around blood vessels in fiber tracts (127). Increased ganglioside GM2 has been observed in hippocampus, thalamus, and hypothalamus along with depletion of ceramides (129). Physiologically longer duration evoked compound action potentials (CAP) have been observed in the corpus callosum at 14 and 30 days post-injury with a depression in unmyelinated fiber amplitudes (117). Gene expression studies have documented altered expression of over 5700 genes in the post-blast brain (118) as well as differences in the hippocampal transcriptome between blast and non-blast models (128). Neuroimaging studies have found region specific decreases in fractional anisotropy (FA) on diffusion tensor imaging (DTI) (116) and increased blood–brain barrier permeability post-blast (127).

## Low-Level Blast Exposure in Animal Models and PTSD

The frequent overlap of TBI and PTSD could represent dual exposures to TBI as well as PSTD-related psychological stressors. Alternatively, TBI might induce PTSD like symptoms if blast damaged brain structures involved in the development of PTSD. Current biological models of PTSD postulate that key frontal and limbic structures, including the prefrontal cortex, amygdala, and hippocampus are involved in PTSD development (137, 138). These models suggest that a key element is an inadequate frontal inhibition of the amygdala, a limbic structure central to controlling fear responses. Exaggerated amygdala responses are thought to heighten responses to psychological threats. A substantial body of functional neuroimaging data is consistent with such models, suggesting that in PTSD there is heightened amygdala activity with decreased hippocampal and orbital frontal activity (137). Damage to the prefrontal cortex by TBI could, therefore, predispose individuals to abnormally sustained responses to psychological stressors.

Animal models should be able to isolate blast effects from psychological stressors and studies have begun to address the behavioral effects of blast in animals across a range of exposures (107, 119, 122, 124, 125, 127, 134, 139–142), although not all studies have been careful to exclude subtle motor or sensory deficits as contributing to apparent behavioral effects. These studies have reported at least short-term effects on anxiety as well as impairments in a variety of learning and memory tasks (107, 119, 122, 124, 125, 127, 134, 139, 141). One study reported impairments in prepulse inhibition immediately after blast exposure, although the changes had mostly recovered 90 days after exposure (142). A few studies have begun to address the issue of dual exposure by examining blast in combination with repeated stress (139) or factors such as transportation stress or anesthesia (140). Studies that have specifically addressed behavioral effects in the context of low-level blast exposure are summarized in Table 2. Abnormalities reflecting impaired memory function have been observed in the Morris water maze (MWZ) (107, 116, 119), novel object recognition (121, 127, 128), Y-maze (127, 128), and passive avoidance (107).

Only a few studies have addressed whether low-level blast exposure alone can induce PTSD-related traits or render animals more sensitive to subsequent PTSD-related stressors. The first of these tested rats that received three 74.5 kPa exposures on a variety of PSTD-related traits several months post-exposure (109). The rats exhibited a variety of PTSD-related traits including increased anxiety in an elevated zero maze and increased acoustic startle. They also showed increased anxiety during a predator scent challenge and an increased cued response in a fear-conditioning paradigm (109).

Subsequently, very similar effects were noted in mice (126). In these studies, mice were exposed to 20–60 psi blast exposures in which the blast wave rather than being allowed to envelope the brain was delivered to a small area on one side of the cranium. While these exposures are in a range that has commonly induced significant CNS pathology in other studies, Heldt et al. (126) reported that using this limited focal exposure, 25–40 psi blasts produced little histological evidence of brain damage and 50–60 psi exposures produced only scattered axonal degeneration. Thus, on pathological grounds, the exposures appeared consistent with low-level blast. Behaviorally, the 25–40 psi blasts produced transient anxiety in an open field. In tests conducted 2–8 weeks after 50–60 psi blast exposures, mice exhibited increased acoustic startle, perseverance of a learned fear response, and an enhanced contextual fear response along with depression-like behavior and diminished prepulse inhibition.

A third study (108) examined non-anesthetized mice exposed to 100 db noise exposures coupled with 2 psi air blasts administered in sessions consisting of 60 exposures over 1 min. The mice exhibited impaired object recognition and anxiety in an elevated O-maze. The mice also spent less time at the edges of an open-field chamber an effect that the authors interpreted as a fear generalization response to the noise/blast exposure having taken place near a chamber wall. No pathology was described to confirm the lack of histological effects and the high frequency of exposure does not model natural human exposures. The levels of exposure are nevertheless within what would be expected to be a low-blast exposure, thus providing additional evidence for cognitive and emotional effects of low-level blast although the lack of anesthesia makes it difficult to determine how much of a psychological stressor may have been involved.

Collectively, the above studies argue that low-level blast exposure alone can induce PTSD-related traits. The increased acoustic startle and altered fear-conditioning responses are of particular interest because of their direct relevance to PTSD (109, 126). The ability of a prior blast exposure to alter subsequent response to a predator scent (109) suggests that blast may render animals more sensitive to later PTSD-related stressors. The fact that the blast overpressure injuries in two of these studies (109, 126) occurred while animals were under general anesthesia, suggests that low-level blast exposures can in the absence of any psychological stressor induce PTSD-related traits. Altered fear responses are suggestive of heightened amygdala function, an effect consistent with current biological models of PTSD (137). Indeed, enhancing fear responses may be a feature of both blast and nbTBI, as recent studies have suggested that increased fear responses can be seen in animal models of nbTBI as well (143, 144).

The studies of blast exposure in rats (109) also found elevation in the amygdala of the protein stathmin 1. Stathmin 1 is highly expressed in the amygdala and mice with null mutations of the stathmin 1 gene are impaired in their ability to lean fear responses (145). Polymorphisms in the stathmin 1 gene have been identified that influence fear and anxiety responses as well as cognitive and affective processing in human beings (146, 147). Heldt et al. (126) also observed in mice reduced numbers of a subpopulation of excitatory projection neurons in the basolateral amygdala that have been linked to fear suppression. Following blast, Xie et al. (108) reported electrophysiological changes in the anterior cingulate cortex, a region implicated in processing emotional memory and inhibitory control. Thus, multiple studies have found blast-associated biochemical, anatomic, or electrophysiological changes in structures relevant to the development of PTSD.

Clearly, questions remain and especially long-term studies are needed to extend these findings. For example, it is not known whether these traits evolve over time. However, the presence of behavioral changes and elevation of stathmin 1, 8 months after blast exposure in one study (109) suggests that blast can induce PTSD-related traits that are chronic and persistent. The relationship to blast may be complex, however, in that another study has suggested that blast exposure causes more rapid extinction of a conditioned fear response if the overpressure injury occurs after learning the conditioned fear response (136).

## Low-Level Blast Exposure in Animal Models and Neurodegenerative Diseases

Recently, nbTBI has been linked to the later appearance of progressive neurodegenerative disorders. The two diseases that have attracted the most concern are Alzheimer’s disease (AD) and chronic traumatic encephalopathy (CTE) (148). Single severe nbTBI earlier in life seems to predispose to the later development of AD. By contrast, repetitive non-blast mTBI has been associated with CTE. The disorders differ in that pathologically AD is characterized by amyloid plaques and tau positive neurofibrillary tangles while CTE is primarily a tauopathy that may have diffuse amyloid plaques but not typically the neuritic plaques characteristic of AD (149). What is now called CTE was first described in boxers as dementia pugilistica. However, CTE has now been seen in a variety of other sports including American football, hockey, soccer, and professional wrestling (39, 150–154).

Whether blast exposure causes or predisposes to later development of neurodegenerative diseases is unknown. Indeed, little is known about long-term effects of blast exposure although one study of 167 U.S. military service personnel who were evaluated within 5 years of sustaining an mTBI in Iraq or Afghanistan found that while many improved, a substantial minority (≈20%) reported new symptoms (155). CTE might be of particular concern with respect to blast due to its association with repetitive mTBI, which has been common in Iraq and Afghanistan and cases of CTE have been reported in veterans returning from these conflicts (122, 156, 157).

Following nbTBI, several proteins associated with neurodegenerative diseases accumulate in brain, including tau, APP, and its product the β-amyloid (Aβ) protein (158). The Aβ peptide is most associated with AD where it deposits in senile plaques with many *in vitro* as well as *in vivo* studies demonstrating that in particular the longer Aβ 42 species can be neurotoxic and associated with a chain of pathological events (159). Interestingly, changes in Aβ occur rapidly after nbTBI in human beings with diffuse cortical Aβ deposits and increased levels of soluble Aβ appearing as early as 2 h after a severe TBI (148, 160–162).

Elevations of Aβ occur consistently in experimental animal models of nbTBI (163–172) along with increased expression of components of the γ-secretase complex as well as BACE1 (β-site APP cleaving enzyme 1), the principal β-secretase (165, 173–176) both of which process APP toward the Aβ pathway. Studies in nbTBI animal models also consistently find elevated APP protein acutely following TBI (176–179). All these observations are consistent with increased processing of APP toward Aβ production after TBI creating interest in whether APP upregulation following TBI may explain the epidemiological association between a history of prior TBI and the subsequent development of AD (148, 160).

Exploring how blast-related TBI affects expression of proteins related to neurodegenerative diseases is just beginning. However, interestingly the one study that has so far reported on Aβ levels following blast found that in both rat and mouse models of blast injury, rather than being increased, brain Aβ levels were decreased acutely following injury (135). In these studies, rats were exposed to single 36.6, 74.5 kPa or 116.7-kPa-exposures and Aβ 40 and 42 levels were examined 24 h and 1 week after exposure. Blast exposure lead to diminished levels of Aβ 42 in rats with the effect on Aβ 42 most prominent in rats exposed to the lowest level blast exposures (36.6 and 74.5 kPa), while no effects on Aβ 42 were seen at the 116.7 kPa exposure level. There were no consistent effects on Aβ 40 levels in rats, with only the 74.5 kPa exposure showing diminished levels 1 week post-exposure. Levels of APP were increased following blast exposure although there was no evidence of axonal pathology based on APP immunohistochemical staining. Unlike in nbTBI animal models, levels of BACE1 and the γ-secretase component presenilin-1 were unchanged following blast exposure in rats. In mice, only a single blast exposure of 147 kPa was tested, an exposure that is more equivalent to a high-level blast exposure (180). Yet, at this exposure, both Aβ 40 and Aβ 42 were decreased 24 h after the blast.

As noted above, multiple studies in experimental animals have found that APP expression increases acutely following TBI (176–179). Indeed, APP accumulation in axons is widely used as a marker of axonal injury in both human beings and experimental animal models of TBI (181–185). However, while one study has reported APP accumulation in axons following blast exposure (186), multiple others have not (107, 113, 115, 118). Risling et al. (113), for example, noted no APP accumulation in axons of rats exposed to 130 and 260 kPa exposures. Garman et al. (115) studied rats exposed to greater than 240 kPa (35 psi) blast exposures and found evidence of widespread diffuse axonal injury by silver staining. However, despite evident axonal injury by silver staining, APP-stained sections typically showed only minimal axonal staining, except for rats studied at 24 h post-exposure when mild axonal staining was described within the deep cerebellar white matter and adjacent to some foci of acute neuronal degeneration. Thus, while APP accumulation in axons is considered a hallmark of acute axonal injury in both human beings and experimental animal models of nbTBI (181–185), it is at most an inconsistent feature of blast-related TBI.

In contrast to the seemingly paradoxical effects of blast on Aβ, multiple studies have found that aberrant tau species are induced by blast (122, 123, 141, 187). Indeed, Goldstein et al. (122) described CTE-linked neuropathology in wild-type C57BL/6 mice 2 weeks after exposure to a single blast. The blast-exposed mice also expressed multiple species of abnormally phosphorylated tau similar to those seen in human neurodegenerative diseases. Huber et al. (123) studying a mouse model exposed to a single 108.9 kPa (15.8 psi) exposure found multiple aberrantly phosphorylated- and cleaved-tau species present at 24 h post-blast that were still present in hippocampus 30 days after exposure.

Thus, experimental animals provide support for a linkage between blast exposure and aberrant tau processing. Lacking are longitudinal studies to determine whether blast induces a CTE-like neurodegenerative disease with progressive features. Indeed, there are few studies on long-term effects of blast and whether effects are static or can be associated with a progressive neurodegenerative disorder although in one study (116) changes in DTI were followed in a rat model exposed to 100 or 450 kPa exposures. Region specific decreases in FA were found at both exposure levels 4 and 30 days post-exposure. Interestingly, the changes evolved during the 4–30-day post-blast period such that a wider area of decreased FA was present at 30 days, changes suggesting an evolving lesion (116).

## Is Blast TBI Different from Non-Blast TBI?

The unexpected lowering of Aβ following blast exposure besides having implications for understanding blast pathophysiology also raises the question of whether blast pathophysiology is different from nbTBI. nbTBI has been so consistently associated with elevated Aβ acutely that decreased levels following blast seem to suggest that blast is pathophysiologically different from nbTBI. The lack of an axonopathy characterized by APP accumulation is also different from nbTBI. These findings have practical implications for treatment of acute blast injury, since blocking Aβ production by a variety of pharmacological or genetic means reduces tissue damage acutely and improves outcome following controlled cortical impact injuries in mice (165, 166, 169). However, such strategies may not be applicable to treatment of acute blast injuries.

Like the issue of longitudinal effects, the question of pathophysiologic distinctness of blast deserves more study although one recent study comparing RNA expression changes between nbTBI and blast TBI models suggests differences as well (128). In these studies, the mouse hippocampal transcriptome was compared following injury using a weight drop model designed to approximate an mTBI or exposure to a 17.2 kPa (2.5 psi) blast. While a common set of upregulated or downregulated RNAs were found, most of the transcriptome changes differed between the two models suggesting that at the molecular level, the TBIs are distinct. Interestingly, in a functional pathway analysis, genes upregulated or downregulated in AD were regulated in similar directions by nbTBI while the opposite was seen following blast with the “Alzheimer’s Disease Up” pathway downregulated by blast and the “Alzheimer’s Disease Down” upregulated by blast (128). Combined with the lowering of Aβ following blast, these studies support the notion that blast-related TBI is pathophysiologically distinct from nbTBI and that in particular multiple pathways related to AD are affected differently by blast and nbTBI.

## Evidence for Effects of Blast-Related mTBI in Human Beings

Close exposure to high-pressure blast waves can cause extensive CNS injury in human beings (47). However, this injury is without doubt a combination of primary, secondary, tertiary, and sometimes quaternary effects in which the role of the primary blast wave itself is hard to define. Determining the role of the primary blast wave in mTBI should be more straightforward since secondary, tertiary, and quaternary effects are less prominent. Yet, as discussed throughout this review, defining the residual effects of blast-related mTBI has been complicated by the frequent presence of co-morbid PTSD.

A number of clinical studies suggest that the link between mTBI and PSTD may be more than coincidental. Mora et al. (95) reviewed records of over 300 patients admitted consecutively to the United States Army Burn Center for explosion-related injuries and examined the prevalence of PTSD in burn patients with and without primary blast injury or mTBI. They found a greater prevalence of PTSD in burn patients with primary blast injury and mTBI than in burn patients injured by other mechanisms. Walilko et al. (96) in a study of 124 survivors of the Oklahoma City bombing explored the relationship between PTSD and physical injuries. They found an association between PTSD and head/brain injuries while PTSD was not highly correlated with other injuries.

Studies of Vietnam veterans have also suggested that TBI is associated with more severe PTSD (188), and in veterans from Iraq and Afghanistan, PTSD is more prevalent in those reporting an mTBI, as compared to veterans who suffered no injury or injuries not involving the head (10). Indeed, in the study of Hoge et al. (10), mTBI was associated with PTSD even after controlling for intensity of combat experience and a recent study of over 27,000 U.S. Army Special Operations Command personnel found that while a blunt or blast-related mTBI increased the chance of reporting clinically significant levels of PTSD symptoms, residual PTSD symptoms were more prevalent in personnel with blast-related mTBI (91). In addition, a dose–response gradient existed between number of blast-related mTBIs and symptom severity (94). Other clinical studies are also consistent with blast-related mTBI, promoting the development of or worsening of PTSD (97–99).

Thus, collectively a body of literature can be seen as arguing that TBI may predispose to the development of or worsen the symptoms of PTSD. However, their interpretation is limited by their observational nature and complicated by the contrary literature already discussed suggesting that most post-concussive symptoms following blast-related mTBI are only non-specifically related to blast or better explained by PTSD (10, 70, 72, 73, 83–91).

Modern neuroimaging would seem ideal for examining the effects of blast-related mTBI (189). By comparing subjects with blast-related TBI/PTSD to those with PTSD alone, it should be possible to determine whether a blast-related signature exists. A number of neuroimaging studies of mostly blast-related mTBI have appeared utilizing mostly DTI, functional MRI (fMRI), or positron emission tomography (PET) scanning with fluoro-deoxyglucose. These studies are summarized in Table 3. Studies range from case reports or small case series to those having over 60 blast-exposed veterans and include some subjects imaged within weeks of the TBI although most involve subjects studied several years after exposure. Some studies are limited by the lack of a military control group and some involve a mixture of blast as well as non-blast injury.

**Table 3:** **Human imaging studies of blast-related TBI**.

Imaging modality	Study subjects	Control group	Findings	Reference
Diffusion tensor imaging	Case study of soldier exposed to a large ordinance explosion.	None; abnormal side was compared to contralateral side.	Reduced FA in the left cerebellar hemisphere 7 months after exposure that normalized on follow-up study.	(190)
Diffusion tensor imaging	37 Iraq and Afghanistan veterans studied on average 871.5 days after mild to moderate TBI.	15 Iraq and Afghanistan veterans without blast exposure who sustained injury to other body regions or had no injury.	No between-group differences in FA and apparent diffusion coefficients across white matter regions known to be vulnerable to axonal injury.	(191)
[^18^F] fluoro-2-deoxyglucose PET (FDG PET)	12 Iraq veterans with at least 1 blast exposure resulting in an mTBI; imaged average of 3.5 years after last exposure.	12 cognitively normal community volunteers without a history of head trauma.	Veterans with blast-related mTBI with or without PTSD showed regional hypometabolism infratentorially (cerebellum, vermis, and pons) and in medial temporal regions.	(192)
Diffusion tensor imaging	63 U.S. military personnel with mTBI evacuated from Iraq or Afghanistan to Landstuhl Germany; all primary blast exposure plus another blast-related mechanism of injury such as struck by blunt object, injured in fall or motor vehicle crash; scanned within 90 days of injury.	21 U.S. military personnel who had blast exposure and other injuries but no clinical diagnosis of TBI.	Reduced FA in veterans with blast-related mTBI in middle cerebellar peduncles, cingulum bundles, and the right orbitofrontal white matter; follow-up scans in 47 subjects 6–12 months later showed persistent abnormalities consistent with evolving injuries.	(193)
Diffusion tensor imaging	25 Iraq and Afghanistan veterans with blast-related mTBI; injuries occurred 2–5 years before imaging.	33 veterans who had not experienced an explosive blast or symptoms of mTBI.	Blast-related mTBI associated with diffuse, global pattern of reduced FA; pattern not affected by history of previous civilian mTBI; with history of more than one blast mTBI trend toward larger number of low FA voxels than with a single blast injury.	(194)
Diffusion tensor imaging	Case report of a marine exposed to multiple primary blasts during a 14-year military career.	A composite fractional-anisotropy template derived from 10 age-matched male veterans without TBI.	Subject had lower FA values in major fiber bundles including the genu, body, and splenium of the corpus callosum and projections that extend bilaterally into the frontal and parietal cortices.	(195)
Diffusion tensor imaging	46 veterans who experienced blast-related mTBI in Iraq and Afghanistan.	None; effects of altered level of consciousness (AOC) vs. loss of consciousness (LOC) and effects of PTSD or major depression examined within subjects.	LOC associated with lower fractional anisotropy (FA) than AOC in 14 regions, including the superior longitudinal fasciculus and corpus callosum; no regions of FA difference between individuals with and without PTSD, or between individuals with and without major depression.	(196)
Diffusion tensor imaging	30 Iraq and Afghanistan veterans who served in combat, and experienced at least one mTBI; imaged approximately 4 years after last tour of duty.	22 Iraq and Afghanistan veterans without a history of TBI.	Blast exposure associated with lower 1st percentile values of FA; lower FA in inferior cerebellar peduncle, fornix, midbrain, and splenium of the corpus callosum before corrections for multiple comparisons.	(93)
High angular resolution diffusion imaging (HARDI)	30 Iraq and Afghanistan veterans with mTBI as well as co-morbid PTSD and depression.	Non-TBI primary (*n* = 42) and confirmatory (*n* = 28) control groups from registry of military service members and veterans who served in Iraq and Afghanistan.	Loss of white matter integrity in primary fibers with mTBI in a widely distributed pattern of major fiber bundles and smaller peripheral tracts including corpus callosum, forceps minor, forceps major, superior and posterior corona radiata, internal capsule, and superior longitudinal fasciculus; loss of white matter integrity correlated with duration of loss of consciousness as well as “feeling dazed or confused” but not with a diagnosis of PTSD or depressive symptoms.	(197)
Task-activated functional MRI (fMRI) (Stop Signal Task)	Iraq and Afghanistan veterans with blast-related mild to moderate TBI (*n* = 21); civilians with TBI from sports or motor vehicle accidents (*n* = 21); injuries occurring 1–6 years before enrollment.	Deployed Iraq and Afghanistan veterans who never experienced blast and/or head injury (*n* = 22); civilians with orthopedic injuries without TBI (*n* = 23).	Different patterns of activation TBI groups vs. controls; pattern of activation different military vs. civilian TBI.	(198)
Diffusion tensor imaging	4 U.S. military personnel deployed to Iraq with primary blast-related traumatic brain injuries; studied 2–4 years post-exposure.	18 US military personnel deployed to Iraq or Afghanistan with no history of head injury, neurological or psychiatric disorders; studied 6–12 months post-deployment.	DTI scans abnormal in 3 of 4 blast TBI subjects; global comparison of relative anisotropy between blast TBI and controls found decrease in relative anisotropy between groups that was driven entirely by findings in the middle cerebellar peduncle.	(199)
Resting-state functional MRI (fMRI)	13 veterans with blast-induced mTBI and no history of blunt head trauma or PTSD.	50 healthy male subjects with no history of head injuries or substance abuse.	mTBI group exhibited hyperactivity in the temporo-parietal junctions and hypoactivity in the left inferior temporal gyrus; abnormal frequencies in default-mode network, sensorimotor, attentional, and frontal networks; functional connectivity disrupted in six network pairs.	(200)
Resting-state functional MRI (fMRI)	63 U.S. military personnel with concussive blast-related TBI; initial scan within 90 days of injury with a follow-up scan 6– 12 months later in a subset of subjects; second independent cohort of 40 U.S. military personnel with concussive blast-related TBI, initial scan within 30 days post-injury.	21 U.S. military controls having blast exposures but no diagnosis of TBI.	Spatially localized reductions in the participation coefficient, a measure of between-module connectivity, in the TBI patients relative to controls at the time of the initial scan; group differences less prominent on follow-up scans; analysis of the second TBI cohort provided partial replication but no substantial differences on the follow-up scans.	(201)
Diffusion tensor imaging; [^18^F] fluoro-2-deoxyglucose PET (FDG PET)	34 Iraq and Afghanistan veterans with a history of one or more combined blast/impact-related mTBI.	18 Iraq and Afghanistan veterans without a history of blast/impact-related mTBI.	Subjects with blast/impact-mTBIs exhibited reduced FA in the corpus callosum; reduced macromolecular proton fraction values in subgyral, longitudinal, and cortical/subcortical white matter tracts and gray matter/white matter border regions; reduced cerebral glucose metabolism in parietal, somatosensory, and visual cortices; neuroimaging metrics did not differ between participants with vs. without PTSD.	(202)
Diffusion tensor imaging	23 veterans of the recent military conflicts exposed to primary blast without TBI symptoms; 6 with mTBI due to primary blast.	16 veterans of the recent military conflicts unexposed to blast.	Lower FA and higher radial diffusivity in veterans exposed to primary blast with or without mTBI relative to blast-unexposed veterans; voxel clusters of lower FA spatially dispersed and heterogeneous across affected individuals.	(203)
Magnetic resonance spectroscopy of the hippocampus at 7 T	25 veterans with mTBI due to blast exposure and memory impairment; all at least 1 year post-exposure.	20 controls not further specified.	Hippocampal *N*-acetyl aspartate to choline and *N*-acetyl aspartate to creatine ratios decreased in comparison to control subjects.	(204)
Diffusion tensor imaging	37 U.S. service members who sustained TBI (29 mild, 7 moderate, 1 severe; 17 blast and 20 non-blast).	14 non-deployed military controls.	Both blast and non-blast TBI reduced FA in multiple white matter tracts; subcortical superior–inferiorly oriented tracts more vulnerable to blast injury than non-blast injury, while direct impact force more detrimental effects on anterior–posteriorly oriented tracts.	(205)
[^18^F]-fluoro-2-deoxyglucose positron emission tomography (FDG PET)	14 Iraq and Afghanistan veterans with a history of blast exposure and/or mTBI.	11 veterans with no history of blast exposure or mTBI	Blast exposure and/or mTBI was associated with lower regional metabolic rates of cerebral glucose consumption during wakefulness and rapid eye movement (REM) sleep in the amygdala, hippocampus, parahippocampal gyrus, thalamus, insula, uncus, culmen, visual association cortices, and midline medial frontal cortices.	(206)
Functional MRI (fMRI) during a pain anticipation task	18 male Iraq and Afghanistan veterans with a history of blast-related mTBI related to combat; studied average 4 years after most severe mTBI.	18 healthy male subjects with no reported history of mTBI	Subjects with a history of mTBI showed stronger activations within midbrain periaqueductual gray, right dorsolateral prefrontal cortex, and cuneus during pain anticipation; effects present after controlling for PTSD and depression.	(207)
Structural MRI	12 active duty service members with blast-related mTBI within last 18 months during deployment to Iraq or Afghanistan.	11 demographically matched control service members without TBI scanned within 18 months of deployment.	Blast injury associated with cortical thinning in the left superior temporal and frontal gyri.	(208)

Despite these limitations, some common findings emerge across studies with the most consistent being reduced fractional anisotropy (FA) on DTI. Indeed, of 11 DTI studies (Table 3), only one failed to find a reduction in FA (191). The patterns across and even within some studies appear fairly heterogeneous and abnormalities have been found in subjects studied out to 4 years post-blast injury. One study using high angular resolution diffusion imaging (HARDI) found suggestions of lost white matter integrity (197). Fewer studies have been conducted with fMRI and PET. However, both resting-state fMRI studies suggested altered patterns of activation (200, 201) and one task-activated fMRI study suggested that activation patterns differed between blast and nbTBI (198). Two PET studies found reduced cerebral glucose metabolism (192, 202).

Thus, a substantial body of evidence suggests that blast-related TBI including blast-related mTBI is associated with a variety of neuroimaging abnormalities that are chronic and persistent. Yet, many subjects in these studies were known to have or even if not mentioned without doubt had PTSD raising the question of whether abnormalities could be attributed to PTSD. Several studies have examined DTI imaging in PTSD (209) although interpreting these studies is often complicated by their inclusion of subjects whose psychological stressor included TBI. Despite these limitations, a recent meta-analysis of seven DTI studies of adult onset PTSD concluded that PTSD is associated with clusters of both increased and decreased FA in various structures (209) suggesting a pattern different from the consistently reduced FA found in blast-related TBI.

Among the blast-related neuroimaging studies, Petrie et al. (202) examined the effect of PTSD on DTI and fluoro-2-deoxyglucose PET imaging in veterans with blast/impact-mTBI. They found areas of reduced FA in the corpus callosum and decreased cerebral glucose utilization in parietal, somatosensory, and visual cortices compared to controls. Their findings did not differ between veterans with vs. without PTSD suggesting that the abnormalities were not related to co-morbid PTSD. Matthews et al. (196) also found no effect of PTSD on FA in a group of Iraq and Afghanistan veterans all of whom had experienced blast-related mTBI. Likewise, Morey et al. (197) using HARDI while finding a widely distributed pattern of lost white matter integrity with blast-related mTBI found no correlation between their findings and a diagnosis of PTSD or depression. More research is needed but based on available evidence it seems unlikely that co-morbid PTSD can account for the neuroimaging changes found in blast-related mTBI.

Whether any of these findings in blast-related mTBI can be extrapolated to subclinical blast where by definition no TBI event occurred is unclear. Studies of subclinical blast are quite limited at present, although interestingly when Taber et al. (203) compared veterans exposed to primary blast with and without mTBI relative to veterans without blast exposure, they found lower FA and higher radial diffusivity in veterans exposed to blast whether or not they had been diagnosed with an mTBI. Mac Donald et al. (210) in a prospective study involving active duty U.S. military personnel evacuated from Iraq or Afghanistan to Landstuhl, Germany while finding no differences in clinical outcomes based on blast vs. non-blast mechanisms of injury found that blast-exposed controls had worse headaches and more severe PTSD than non-blast-exposed controls. Using data from a DTI study, Bazarian et al. (93) have also argued that subclinical blast may play a role in the genesis of PTSD.

Clearly, future studies directed at in particular neuroimaging of subclinical blast will be important although study of this problem in active duty military personnel where exposures are often a combination of clinically recognized and unrecognized events may be difficult. Among groups that might be studied for effects of subclinical blast, breachers provide perhaps the most accessible population where blast exposure in human beings can be studied in a semi-controlled manner. “Breachers” are a population of military and law enforcement personnel who are routinely exposed to low-level blast during their training and in the course of operations. Repeated exposure in these settings has been associated with symptoms similar to those seen with sports concussion (211). Recently, Tate et al. (211) examined the effects of repeated low-level blast exposure without mTBI during an explosives training course in 21 members of the New Zealand Defense Force. Self-reported symptoms, neurocognitive performance, and three serum biomarkers (ubiquitin C-terminal hydrolase-L1, the alpha II-spectrin breakdown product, and glial fibrillary acidic protein) were measured before, during, and after a 2-week course. Interestingly, those with the highest biomarker loads had longer reaction times, lower cognitive test scores, and reported more symptoms than those with the lowest biomarker loads. Preliminary studies have also observed alterations in white matter and cortical structural MRI measures in a cohort of breachers with 7–21 years of prior blast exposure that were not seen in a group of students attending a 2-week breacher course (212). Indeed, breachers [and similar populations who are exposed to repetitive blast events, e.g., artillery, explosive ordinance disposal, shoulder-fired weapons, etc.] may represent a unique population in which to study how subclinical blast affects human beings.

## Conclusion

It has long been appreciated that high-pressure blast waves can cause extensive CNS injury in human beings. Less clear are the effects of lower level blast exposures, the most common exposure in combat settings such as Iraq and Afghanistan. Indeed, the traits that can be attributed to blast-related mTBI have been questioned with suggestions that much of what is presently being called post-concussion syndrome secondary to blast-related mTBI is really PTSD. No doubt there are grounds for controversy and many questions remain. Both PTSD and the post-concussion syndrome are clinical diagnoses made in disorders that have overlapping symptoms. Current definitions have lowered the threshold for diagnosing mTBI leading to questions as to whether we are now over diagnosing mTBI. Soldiers that have experienced blast-related mTBI have typically been exposed to psychological stressors as well and there are no biomarkers that can distinguish cognitive, affective, and somatic symptoms induced by a psychological stressor from those induced by physical trauma. PTSD is a well-established clinical syndrome while the effects of human low-level blast exposure including mTBI are still being established. Thus, there is much ground for legitimately questioning the importance of low-level blast exposure.

Animal models would seem ideal for determining the effects of primary blast free of many confounding variables present in natural human exposures. Indeed, a substantial literature now exists concerning the effects of blast in animal models. Yet, it must be admitted that this literature is also troubled by variability in experimental approach as well as at times lack of attention to modeling clinically relevant exposures or reliance on models that fail to isolate the effects of primary injury from tertiary injury. There has also been little attention given to defining what “mild” TBI is in an animal model and studies often describe models as “mild” TBI without any justification for why use of the term “mild” is appropriate.

Yet, despite these concerns, an abundance of evidence now supports the concept that low-level blast has significant long-term effects on the nervous system. While problems exist in applying definitions of human mTBI to animal models, conditions of low-level blast exposure can be defined that likely approximate human mTBI or subclinical exposure. In animals, blast exposures in these ranges exert a variety of biochemical, pathological, and physiological effects on the nervous system. Several studies in animals suggest that low-level blast exposure can induce PTSD-related behavioral traits in the absence of a psychological stressor. Indeed, if blast injury can induce PTSD like symptoms without a psychological stressor, then human cases that are presently being labeled PTSD may in fact be part of the spectrum of blast-related brain injury. Several observations in animal models also suggest that blast-related TBI is pathophysiologically distinct from nbTBI. A substantial body of neuroimaging data shows that blast-related mTBI in human beings is associated with chronic decreases in fractional anisotropy that are suggestive of chronic axonal injury and unlikely to be explained on the basis of co-morbid PTSD. In summary, while many questions remain concerning how blast overpressure waves affect the brain, there seems little doubt that low-level blast exposure should be of significant concern.

## Author Contributions

All authors participated in the collection, review, and analysis of the relevant literature as well as the drafting and revising of the manuscript.

## Conflict of Interest Statement

The authors declare that the research was conducted in the absence of any commercial or financial relationships that could be construed as a potential conflict of interest.
